# Transcriptomic and Physiological Analysis Reveals Genes Associated with Drought Stress Responses in *Populus alba* × *Populus glandulosa*

**DOI:** 10.3390/plants12183238

**Published:** 2023-09-12

**Authors:** Tae-Lim Kim, Hyemin Lim, Michael Immanuel Jesse Denison, Changyoung Oh

**Affiliations:** 1 Department of Forest Bioresources, National Institute of Forest Science, Suwon 16631, Republic of Korea; ktlmi01@korea.kr (T.-L.K.); happyohcy@korea.kr (C.O.); 2 3BIGS Company Limited, Hwaseong 18469, Republic of Korea; michael@3bigs.com

**Keywords:** drought stress, physiological response, poplar, transcriptome

## Abstract

Drought stress affects plant productivity by altering plant responses at the morphological, physiological, and molecular levels. In this study, we identified physiological and genetic responses in *Populus alba* × *Populus glandulosa* hybrid clones 72-30 and 72-31 after 12 days of exposure to drought treatment. After 12 days of drought treatment, glucose, fructose, and sucrose levels were significantly increased in clone 72-30 under drought stress. The Fv/Fo and Fv/Fm values in both clones also decreased under drought stress. The changes in proline, malondialdehyde, and H_2_O_2_ levels were significant and more pronounced in clone 72-30 than in clone 72-31. The activities of antioxidant-related enzymes, such as catalase and ascorbate peroxidase, were significantly higher in the 72-31 clone. To identify drought-related genes, we conducted a transcriptomic analysis in *P. alba* × *P. glandulosa* leaves exposed to drought stress. We found 883 up-regulated and 305 down-regulated genes in the 72-30 clone and 279 and 303 in the 72-31 clone, respectively. These differentially expressed genes were mainly in synthetic pathways related to proline, abscisic acid, and antioxidants. Overall, clone 72-31 showed better drought tolerance than clone 72-30 under drought stress, and genetic changes also showed different patterns.

## 1. Introduction

Drought, a significant abiotic environmental factor globally, hinders plant growth and production. Consequently, developing drought-tolerant varieties has become a primary objective for researchers in plant cultivation. Drought stress, an unpredictable stressor, directly or indirectly impairs nearly all stages of a plant’s life cycle [1,2,3]. It influences carbon assimilation, gaseous exchange, and oxidative damage, leading to abnormal physiological processes that negatively impact productivity [4,5]. Furthermore, it results in a yield decline in tree crops due to diminished leaf development, reduced enzyme activity, and decreased ion absorption [2,3,6]. Numerous studies have recently reported on the effects of drought stress and plant adaptation. These effects include alterations in crop yields, growth, pigment synthesis, photosynthesis activity, membrane integrity, osmosis regulation, pore opening, cell division, and the accumulation of reactive oxygen species (ROS) [7,8,9]. Plants can bolster their drought resistance by enhancing the activities of superoxide dismutase (SOD), peroxidase (POD), and catalase (CAT), increasing lignin content, and reducing malondialdehyde (MDA) and ROS contents [10,11,12]. Additionally, the physiological drought response involves the accumulation of osmotic substances [13] and changes in phytohormone and chlorophyll content [14]. Osmotic substances in plants include proline, betaine, trehalose, fructose, and inorganic ions. Among these, the most recognized plant hormone, abscisic acid, has intricate mechanisms and plays a crucial role in responding to drought stress [15,16]. Changes in MDA content, epidermal cells, and vascular tissue cells are also known to occur under abiotic stress [17,18]. Research on molecular mechanisms conferring drought resistance has been conducted in *Arabidopsis*, rice, and grasses. These studies have discovered that protein kinases, transcription factors, and protein phosphatases play a role in drought resistance [14].

Poplar is an important woody crop that contributes to bioenergy production and carbon sequestration due to its substantial biomass. It is important both ecologically and economically [19]. In addition, poplar’s rapid growth rate has led to its recognition as an economically valuable tree. As such, it has recently become a target species for research into genotypes, transcriptomes, and drought response mechanisms [20,21]. The availability of the poplar genome is crucial for understanding the molecular processes involved in growth, metabolism, and responses to environmental perturbations. Poplar is extensively used in greening, afforestation, and production due to its rapid growth, strong adaptability, and superior material quality. Poplar is often planted in marginal areas where water and nutrient resources are limited [22,23]. The drought response in *Populus* species involves growth inhibition, increased ABA levels, and altered anatomical properties [23,24].

RNA sequencing is a crucial method for examining gene expression in certain tissues at certain points in time [25]. RNA transcriptomics constitutes a potent method that allows for variant detection, gene-specific expression, genome-wide transcript characterization, and differential gene expression analysis. These methods enhance our comprehension of genetic variation in complex phenotypic traits such as drought tolerance and deepen our understanding of drought stress response pathways [26]. Transcriptomic studies have enabled the analysis of genes that respond to drought stress and are involved in plant regulatory mechanisms [1,27]. Specifically, in *Arabidopsis thaliana*, thousands of genes are thought to play a role in drought, cold, and salt stress [28]. These studies suggest that oxidation-reduction processes are implicated in the mechanisms of many salt tolerance genes that encode proteins involved in osmotic protection metabolism, ion transport, heat shock proteins, and hormone signaling. Therefore, the identification of gene expression related to drought tolerance remains a critical aspect of tree breeding programs and aids in pinpointing key genes involved in drought tolerance. The candidate genes identified are crucial for the development of resistant varieties.

Thus, the objective of this study was to examine the physiological and transcriptional alterations in poplar subjected to drought stress. We aimed to correlate these physiological parameters with shifts in transcript abundance.

## 2. Results

### 2.1. Growth and Physiological Response to Drought Stress in Poplar

**Figure 1:**
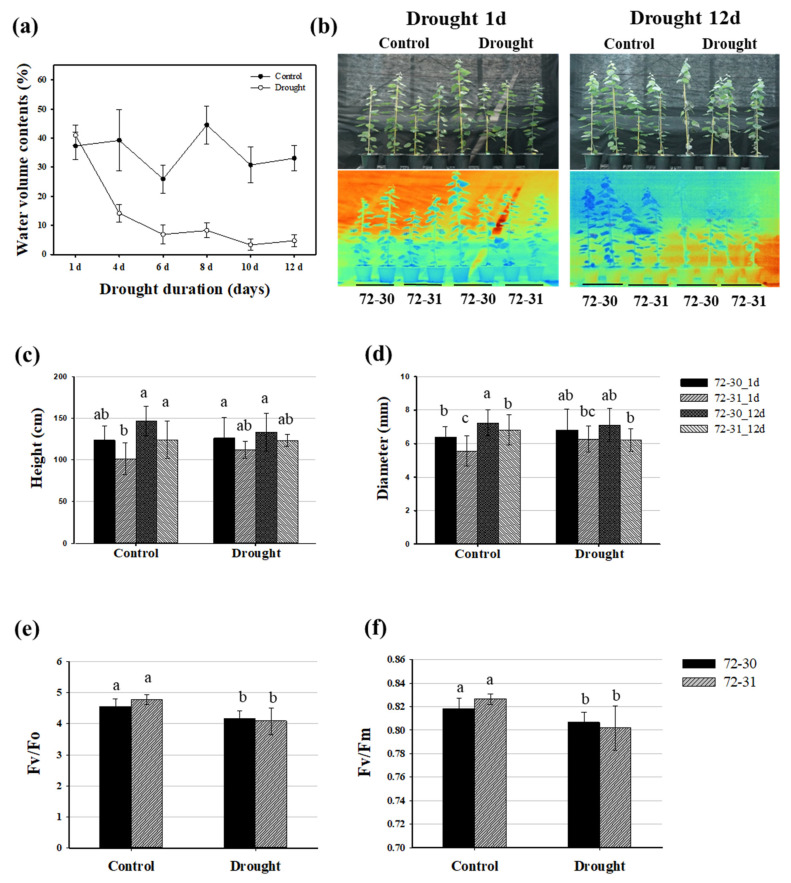
The growth phenotype and physiological changes of *P. alba* × *P. glandulosa* clones 72-30 and 72-31. (**a**) Volumetric water content in the soil of drought-treated plant pots. (**b**) Infrared thermal images. The effect of drought treatment on the shoot growth (**c**) and diameter (**d**) of plants. Chlorophyll a fluorescence as an indicator of the response to drought treatment. (**e**) Fv/Fo is represented as a ratio. (**f**) Fv/Fm is represented as a ratio. The values are the means ± SDs (*n* = 9). Different lowercase letters indicate significant differences (ANOVA with Tukey’s HSD, *p* < 0.05).

To investigate the growth and physiological responses of *P. alba* × *P. glandulosa* to water deficits, the 72-30 and 72-31 hybrid clones were assessed after 1 and 12 days of drought treatment. We compared the soil moisture content of the drought-treated plants with that of the control group throughout the treatment period. After 12 days of drought treatment, the soil moisture content decreased to less than 2% (Figure 1a). We observed the growth phenotype using infrared (IR) digital thermal imaging after 12 days of drought treatment. The temperature of the drought-treated poplar leaves was approximately 0.6 °C higher than that of the control group (Figure 1b). Although there were no statistically significant differences between the control and drought stress conditions, the leaves of both clones tended to wither, and their height and diameter appeared to decrease under water deficit conditions (Figure 1b–d). We measured the photochemical efficiency of photosystem II (PSII) under the Fv/Fm condition and found that it decreased in both 72-30 and 72-31 clones after drought treatment. The potential activity of PSII, as indicated by the Fv/Fo ratio, also significantly decreased in both clones with drought treatment. The Fv/Fo of 72-30 and 72-31 clones decreased by 8.3% and 10.4%, respectively, compared to the controls. This decrease in PSII activity could be due to moisture deficiency (Figure 1e,f). Additionally, leaf chlorophyll content is often used as an indicator of photosynthetic activity, and it can be affected by abiotic stresses such as drought. After drought treatment, the contents of chlorophyll a, b, and total in the 72-31 clone significantly increased. However, there was no significant change in the chlorophyll content of the 72-30 clone under drought-stress conditions (Table 1). Furthermore, the 72-31 clone showed increased carotenoid content after drought treatment.

### 2.2. Effect of Drought Stress on Soluble Sugars, Lipid Peroxidation, Proline, H_2_O_2_, and Antioxidant Activities

To investigate the effects of drought stress on the stress-responsive elements, carbohydrates and antioxidants were measured. Carbohydrate levels were analyzed in the leaves of 72-30 and 72-31 clones to investigate the effect of drought stress on carbon partitioning. The major carbohydrates detected in the leaves of poplars were glucose, fructose, and sucrose. Clone 72-30 showed significant increases in glucose, fructose, and sucrose contents after drought treatment (Table 2). Glucose, fructose, and sucrose contents of clone 72-30 exhibited a significant increase after drought treatment, while starch was no significant change. In clone 72-31, glucose and fructose significantly increased under drought conditions, while the starch content was reduced by half. 

**Figure 2:**
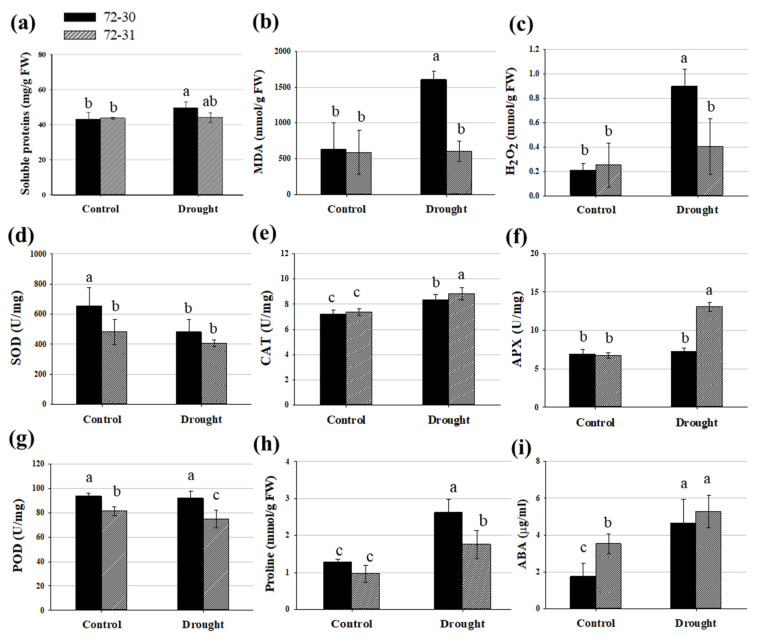
Effect of drought stress on drought stress indicators and antioxidants. (**a**) Soluble protein content. (**b**) MDA content. (**c**) H_2_O_2_ content. (**d**) SOD activity. (**e**) CAT activity. (**f**) APX activity. (**g**) POD activity. (**h**) Proline content. (**i**) ABA content. Different lowercase letters indicate significant differences (ANOVA with Tukey’s HSD, *p* < 0.05).

Soluble proteins significantly increased by about 15% in the 72-30 clone under drought stress (Figure 2a). The MDA content was used as a classic marker of membrane lipid peroxidation and tissue damage induced by drought stress. After the drought treatment, the MDA content of the 72-30 clone exhibited was significantly increased (Figure 2b). However, the MDA content in the 72-31 clone did not show any statistically significant change. Experiments were conducted to investigate changes in H_2_O_2_ levels in leaves under drought stress in order to determine the production of H_2_O_2_ due to drought (Figure 2c). The 72-30 clone had a significantly increased concentration of H_2_O_2_ in its leaves. Overall, the 72-30 clone had approximately twice the H_2_O_2_ content of the 72-31 clone.

Representative antioxidants include SOD, CAT, APX, and POD, and the activities of these enzymes were measured (Figure 2d–g). In clone 72-30, there was a decrease in SOD activity, an increase in CAT activity, and no change in POD activity compared to the control. Conversely, in clone 72-31, there was a significant increase in CAT and APX activity and a decrease in POD activity.

We assessed the proline content in hybrid poplar clones, finding it to be relatively high when compared to the control (Figure 2h). Proline, a key indicator of drought stress, showed an increase in concentration across both clones, with a particularly notable doubling in clone 72-30. We also evaluated the changes in ABA content before and after the application of drought treatment. The ABA content rose by over 170% in clone 72-30 and approximately 48.5% in clone 72-31 (Figure 2i).

### 2.3. RNA Sequencing, Assembly, and Annotation

Twelve RNA-seq libraries generated a total of 0.346 billion raw reads. The number of raw reads per sample ranged from 21.3 to 33.0 million, with an average of 28.9 million. After filtering and trimming, the number of high-quality clean reads per sample ranged from 19.4 to 30.5 million, with an average of 26.6 million. More than 95% of the clean reads in all libraries had high quality at the Q30 level, and more than 92% of the clean reads were assigned to the *P. alba* reference genome. The GC concentration of the samples was similar, ranging from 46% to 50%. The average percentage of GC was 47.8% (Table 3). Subsequently, the mapped reads were assembled and quantified using StringTie. In total, 46,829 genes were identified from all samples that had FPKM values greater than 0 and were considered expressed.

### 2.4. Analysis of Differentially Expressed Genes in the Response to Drought Stress

**Figure 3:**
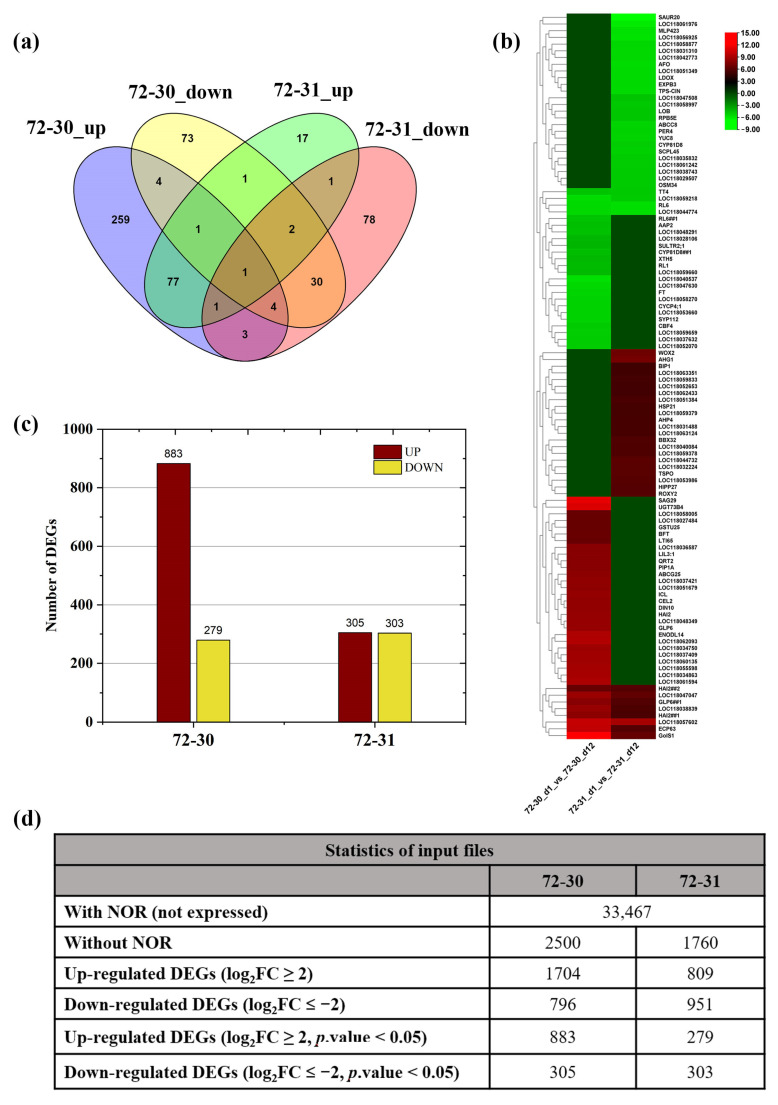
Statistics of differentially expressed genes (DEGs) in the two comparison groups from RNA-seq data. Analysis of differentially expressed genes in *Populus* under drought stress conditions. (**a**) Venn diagram representing the numbers of DEGs. (**b**) Top 30 up-regulated and down-regulated genes between two comparisons. (**c**) Number of DEGs in the two groups. (**d**) Tabular data showing the complete statistics of the DEGs regulated in the two groups. NOR represents “Non-regulated”.

Differential gene expression studies were performed using the EdgeR package, comparing hybrid clones 72-30 and 72-31 from day 1 and day 12 under drought conditions with a water deficit. Differentially expressed genes (DEGs) were identified based on a log2FoldChange ≥2 between two conditions and a false discovery rate of ≤0.05. When 72-30_d1 and 72-30_d12 were compared, 1188 significant DEGs were detected, of which 883 were up-regulated and 305 were down-regulated. When comparing hybrid clones 72-31_d1 and 72-31_d12, 582 significant DEGs were detected, of which 279 were up-regulated and 303 were down-regulated. The identified DEGs were aligned with the protein database of the reference *P. alba* genome using the BLASTX program in the OmicsBox v2.0.36 software package (Valencia, ES-Spain). Unique and common genes that were regulated in the comparison between 72-30_d1 and 72_30_d12 (351 up-regulated and 116 down-regulated) and in the comparison between 72-31_d1 and 72_31_d12 (101 up-regulated and 120 down-regulated) were identified. Venn analysis showed that 259 DEGs and 73 DEGs were clearly up-regulated and down-regulated, respectively, in a comparison between 72-30_d1 and 72-30_d12. In contrast, only 17 DEGs and 78 DEGs were specifically regulated in the 72-31_d1 versus 72-31_d12 comparison (Figure 3a). Among the DEGs that were specifically up-regulated in a comparison between 72-30_d1 and 72-30_d12, certain drought stress-related genes, such as *NACs*, *DREBs* such as *DREB19*, *DREB2A*, and *DREB2C,* various *ERFs*, *MYB* factors, *PP2s*, and *WRKY* were significant, whereas among the down-regulated DEGs, *MYBs*, *DREBs*, *PYL*, and *ZFP* were significant, but at lower levels. DEGs specifically up-regulated in the 72-31_d1 versus 72-31_d12 comparison were not detected, whereas *MAPKK15*, *MYB* factors, *WRKY6*, and *ZFP6* genes related to drought resistance were down-regulated at lower numbers. A heatmap representation of the top 30 up-regulated and down-regulated genes in the two-hybrid clone comparisons, 72-30_d1 versus 72-30_d12 and 72-31_d1 versus 72-31_d12 is shown in Figure 3b. The number of significantly up-regulated and down-regulated DEGs observed in both comparisons was calculated (Figure 3c,d) (Table 4 and Table 5).

### 2.5. Functional Enrichment of DEGs in the Response to Drought Stress

**Figure 4:**
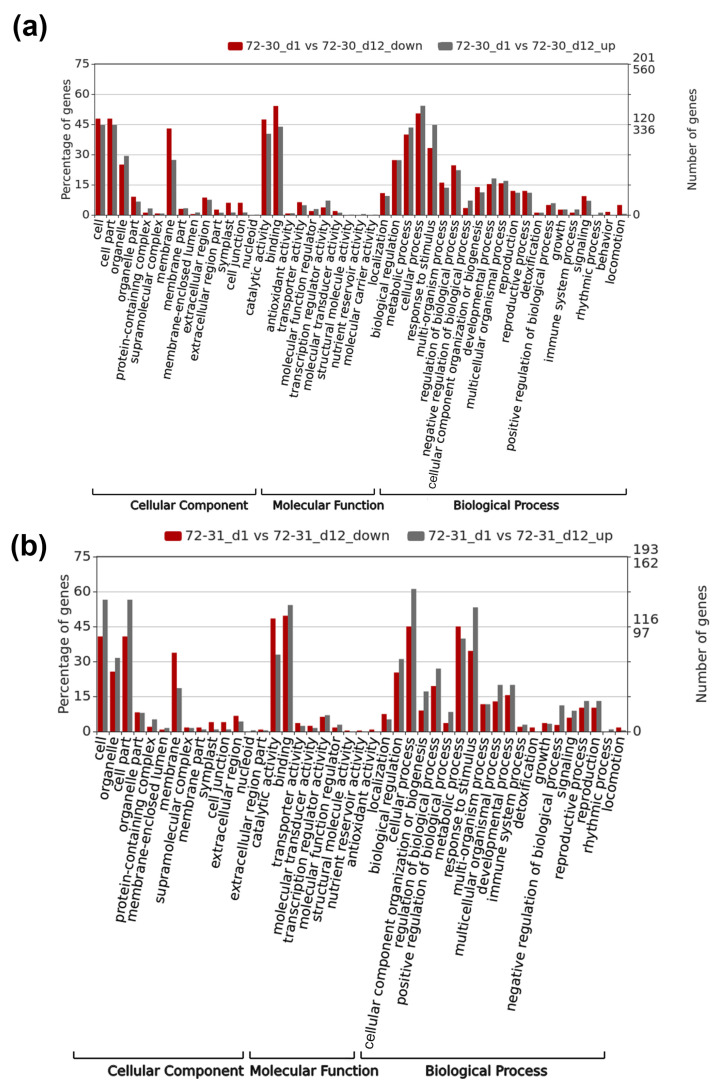
GO categories of *P. alba* × *P. glandulosa* all-unigenes. WEGO was used to create the graph. The results are summarized under the categories of “biological process’”, “cellular component”, and “molecular function.” The percentage (left y-axis) and total number (right y-axis) of all-unigenes in each category (the third level of GO) are shown. The y-axis is scaled in log (10). (**a**) 72-30_d1 versus 72-30_d12. (**b**) 72-31_d1 versus 72-31_d12.

GO enrichment was used to identify the major functional groups of DEGs impacted by drought stress. In the GO classification, 45 overrepresented groups were identified in the 72-30_d1 versus 72-30_d12 comparison, while 43 groups were discovered in the 72-31_d1 versus 72-31_d12 comparison (Figure 4). In the 72-30_d1 versus 72-30_d12 group, the “response to stimulus” category (33%) within biological processes encompassed response to stress (19%) and response to abiotic stimuli (6%). Additionally, 1.9% of the genes were categorized under “response to water deprivation” (Figure 4a). These genes included putative conserved domains such as glycoside hydrolase domains, PP2C domains, and aldoketo reductase domains. In the 72-31_d1 versus 72-31_d12 groups, the “response to stimulus” category (34.5%) within the “biological process” included “response to stress” (19.8%) and “response to abiotic stimulus” (11.6%). Genes corresponding to “response to water deprivation” (1.6%) were also noted (Figure 4b). Upon analyzing both groups, the following genes were found to be particularly relevant in the context of water deprivation in poplar hybrid clones: 11 kDa late embryogenesis abundant protein, 9-cis-epoxycarotenoid dioxygenase NCED1, chloroplastic-like, beta-amylase 1, chloroplastic-like, galactinol synthase 1-like, galactinol synthase 2-like, homeobox-leucine zipper protein ATHB-12-like, homeobox-leucine zipper protein ATHB-7-like, NADPH-dependent aldoketo reductase, chloroplast-like, ninja family protein AFP2-like, protein phosphatase 2C 37-like, REF/SRPP-like protein At1g67360, and an uncharacterized protein C24B11.05-like. 

**Figure 5:**
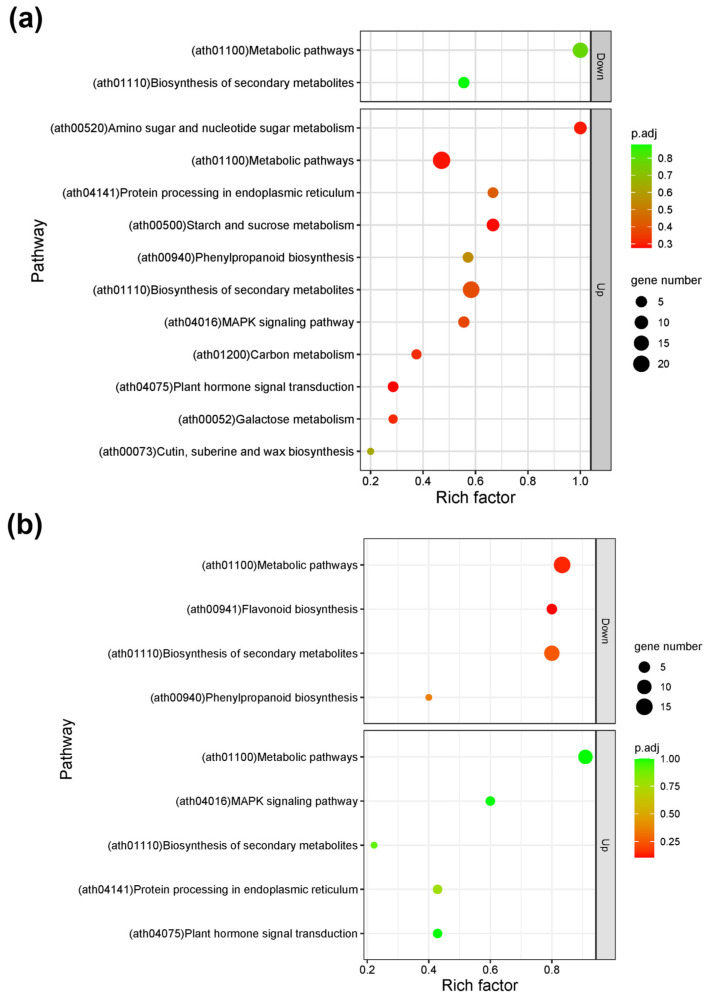
Dot plot of KEGG pathways enriched among differentially expressed genes (DEGs) in (**a**) 72-30_d1 versus 72-30_d12, (**b**) 72-31_d1 versus 72-31_d12. The x-axis shows the rich factor of differentially expressed genes in each term relative to the total number of genes in that term. The y-axis shows the KEGG pathway terms. The size of the dots shows the number of DEGs in that term, and the color is the test statistic of the gene set enrichment analysis with false discovery rate adjustment.

When comparing 72-30_d1 with 72-30_d12, a total of 105 DEG hits were observed in the KEGG database. Of these, 84 were upregulated, and 21 were downregulated. In a similar comparison between 72-31_d1 and 72-31_d12, we noted a total of 54 DEG hits, with 21 upregulated and 33 downregulated in relation to the KEGG pathway of *A. thaliana*. The primary KEGG pathways encompassed MAPK signaling (ath04016), plant hormone signal transduction (ath04075), secondary metabolite biosynthesis (ath01110), and metabolic pathways (ath01100) (Figure 5). 

### 2.6. DEGs Involved in Proline, Antioxidants, and ABA

**Figure 6:**
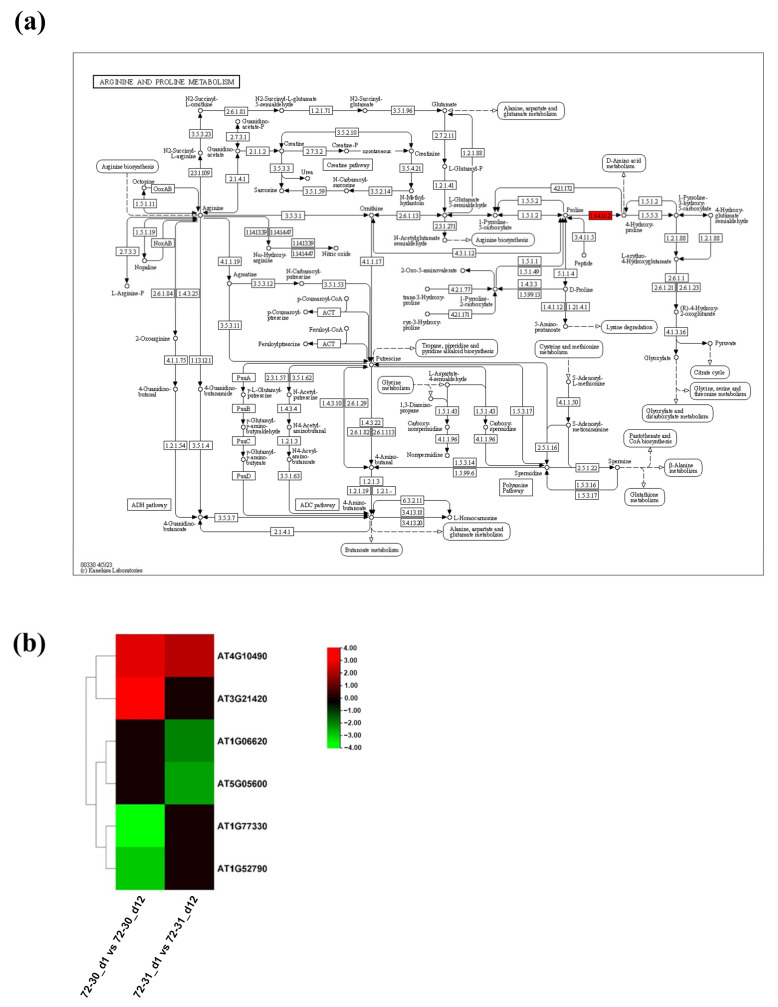
(**a**) KEGG pathway of proline pathways. The red shaded genes indicate the up-regulated genes involved in proline metabolism. (**b**) Heat map showing expression changes in proline.

**Figure 7:**
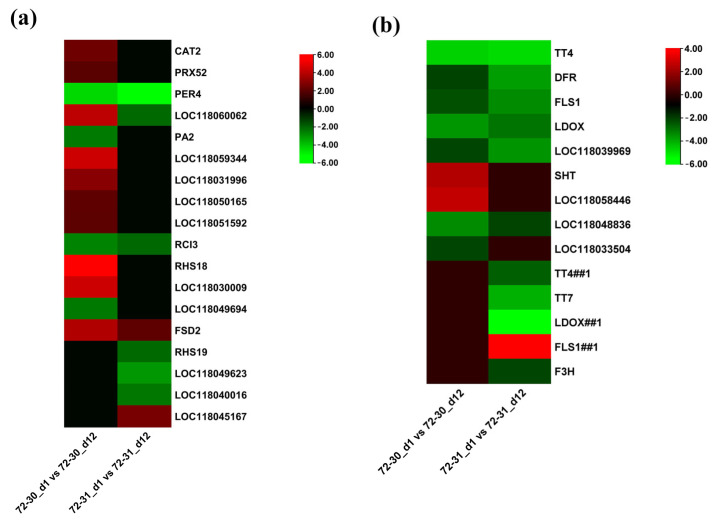
(**a**) Heat map showing expression changes in antioxidants (enzymatic). (**b**) Heat map showing expression changes in antioxidants (non-enzymatic).

**Figure 8:**
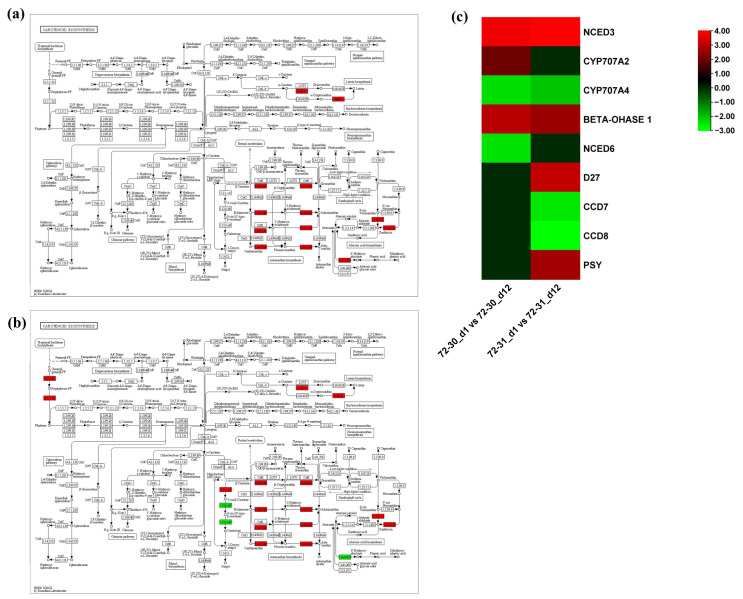
KEGG pathway enrichment of the plant hormone abscisic acid. (**a**) 72-30. (**b**) 72-31. In both (**a**,**b**) red shaded genes were Upregulated while green ones were downregulated (**c**) Heat map showing the differential expression of genes related to abscisic acid. The color bar shows a scale for log_2_ fold changes.

Genes involved in proline metabolism, antioxidant metabolism, and abscisic acid metabolism pathways were tested for their involvement in drought stress in poplar hybrid clones. In the proline metabolism pathway, we observed a significant up-regulation of the genes LBO1 and LOC118040847, while other genes, including LOC118060471 and LOC118034855, were down-regulated in the 72-30_d1 compared to the 72-30_d12 (Figure 6a,b). Additionally, DEGs such as LOC118037479 and LOC118049366 were downregulated in the 72-31_d1 versus 72-31_d12 comparison. The two up-regulated genes in the 72-30_d1 versus 72-30_d12 comparison, LBO1 and LOC118040847, correspond to 2-oxoglutarate (2OG) and Fe(II)-dependent oxygenase superfamily protein. These proteins are crucial for proline metabolism in higher plants, particularly for the oxidation of proline to hydroxy-proline, which results in the accumulation of H_2_O_2_ (https://doi.org/10.1046/j.1365-3040.1998.00309.x, page 541, accessed on 20 June 2023). This finding prompted us to analyze the DEGs involved in the regulation of antioxidant metabolism. In the enzymatic pathway, a majority of genes, including CAT2, PRX52, LOC118060062, LOC118059344, LOC118031996, LOC118051592, RHS18, and LOC118030009, were significantly upregulated in the 72-30_d1 versus 72-30_d12 comparison (Figure 7). In the 72-31_d1 versus 72-31_d12 comparisons, RHS19, LOC118049623, and LOC118040016 were downregulated, while LOC118045167 was upregulated. In the non-enzymatic pathway, genes such as SHT and LOC118048836 were upregulated in the 72-30_d1 versus 72-30_d12 comparison, whereas FLS1 was upregulated in the 72-31_d1 versus 72-31_d12 comparison. We also mapped DEGs regulated in the abscisic acid pathway (Figure 8). This revealed that some genes, including CYP707A2, were upregulated in the 72-30_d1 vs. 72-30_d12 comparison, while D27 and PSY were upregulated and CCD7 and CC8 were downregulated in the 72-31_d1 vs. 72-31_d12 comparison. In both clones, NCED3 and BETA-OHASE1 showed a significant increase in expression, while CYP707A4 was significantly decreased. There was no difference in the expression levels of NCED3, CYP707A4, and BETA-OHASE1 between clone 72-30 and clone 72-31.

### 2.7. Validation of RNA-Seq Results by qPCR

**Figure 9:**
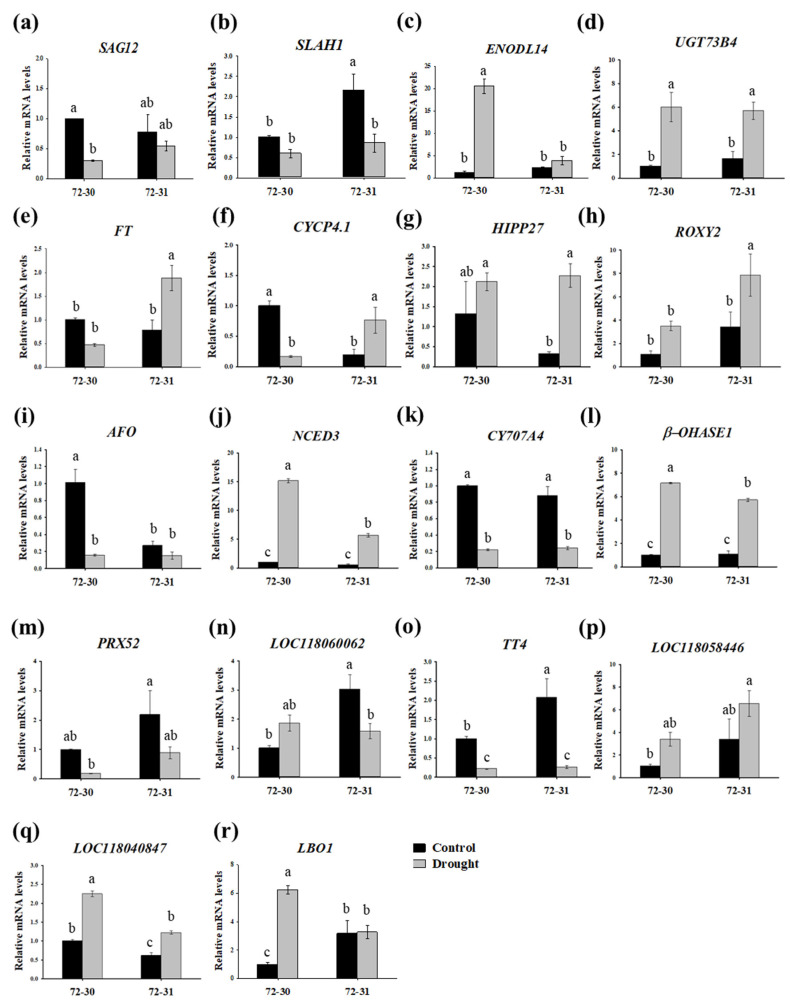
Validation of the differential expression of 18 genes by qPCR. (**a**) Senescence-specific cysteine protease SAG12-like (*SAG12*). (**b**) S-type anion channel SLAH1-like (*SLAH1*). (**c**) Umecyanin-like (*ENODL14*). (**d**) Zeatin O-xylosyltransferase-like (*UGT73B4*). (**e**) Protein HEADING DATE 3A-like (*FT*). (**f**) Cyclin-U4-1-like (*CYCP4.1*). (**g**) Heavy metal-associated isoprenylated plant protein 27 (*HIPP27*). (**h**) Glutaredoxin-C1-like (ROXY2). (**i**) Axial regulator YABBY 1-like (*AFO*). (**j**) 9-cis-epoxycarotenoid dioxygenase NCED1 (*NCED3*). (**k**) Abscisic acid 8′-hydroxylase CYP707A1-like (*CYP707A4*). (**l**) Beta-carotene hydroxylase 2 (*BETA-OHASE 1*). (**m**) Peroxidase 2-like (*PRX52*). (**n**) Lignin-forming anionic peroxidase-like (LOC118060062). (**o**) Chalcone synthase 1-like (*TT4*). (**p**) Stemmadenine O-acetyltransferase-like (LOC118058446). (**q**) Protein DMR6-LIKE OXYGENASE 2-like (LOC118040847). (**r**) Protein SRG1(*LBO1*). qPCR data were analyzed using the 2^−ΔΔCt^ method with *ACTIN* and *UBQ7* as internal controls. Three biological replicates were performed for each sample. Error bars show the standard error of the mean (*n* = 3). The letters a, b, and c indicate significant differences according to ANOVA with Tukey’s HSD test (*p* < 0.05).

To verify the gene expression profiles identified in RNA sequencing results, we conducted qPCR on 18 DEGs from various functional categories, including plant hormone signaling, proline metabolism, and the antioxidant pathway. The expression patterns of all 18 selected DEGs in *P. alba × P. glandulosa*, 72-30 and 72-31, were consistent with those observed in the RNA-seq data (Figure 9), thereby confirming the reliability of the RNA-seq results. The expression of *SAG12* (LOC118062568) and *SLAH1* (LOC118032010), which are overlapping genes of the two clones, was confirmed. We also performed qPCR for *ENODL14* (LOC118052105) and *UGT73B4* (LOC118053155), which were significantly up-regulated, and *FT* (LOC118043148) and *CYCP4.1* (LOC118042489), which were significantly down-regulated only in the 72-30 clone. In clone 72-31, *HIPP27* (LOC118029876) and *ROXY2* (LOC118055294) were the genes with the most up-regulated expression, while *AFO* (LOC118035984) was the most down-regulated. The ABA hormone signaling genes *NCED3* (LOC118038286) and *β-PHASE 1* (LOC118030260) were up-regulated, and conversely, *CYP707A4* (LOC118035767) was down-regulated in both clones, respectively. The antioxidant pathway DEGs, except the up-regulated LOC118058446, *PRX52* (LOC118035629), LOC118060062, and *TT4* (LOC118054546), were down-regulated in 72-30 and 72-31. Regarding genes related to proline biosynthesis, LOC118040847 and *LBO1* (LOC118034519) were up-regulated. These qPCR analysis results were consistent with the RNA-seq results.

## 3. Discussion

Drought is one of the most damaging abiotic stressors for plants and is triggered by insufficient rainfall, rising temperatures, and lack of water availability. It is becoming an increasing problem due to global climate change. Here, we examined the drought response patterns at the transcriptome level in Poplar hybrid clones. We started with potting soil that had a moisture content exceeding 40%. By day 6, we had reduced this to 10% or less, and by the end of the experiment, it was 2% or less. This process was designed to induce a water deficit in the plants (Figure 1a). As a result, we observed a decrease in plant growth and a temperature increase of 0.6 °C compared to the control group, which continued to water their plants (Figure 1b). Additionally, we noted a reduction in the fluorescence response of Fv/Fo and Fv/Fm (Figure 1e,f). Chlorophyll fluorescence can indicate damage to photosynthetic systems or effects related to photoprotection under drought stress [29]. Our study investigated the effects of drought stress on the photochemical efficiency of PSII in two different clones. We employed photosynthesis-related spectroscopy analysis to evaluate the response. It is well-established that water deficit significantly contributes to the reduction in PSII activity [30]. The observed decrease in Fv/Fm in clone 72-30 and clone 72-31 indicates damage to the photosynthetic apparatus due to drought stress. In our analysis, we analyzed the parameter Fv/Fo, which signifies the ratio of photochemical and nonphotochemical de-excitation fluxes of excited chlorophyll. The results suggested a variation in the rate of electron transport from PSII to the primary electron acceptors, which could be influenced by the density and size of the samples [31]. Furthermore, we assessed Fv/Fm, which represents the maximum yield of primary photochemistry and reflects the photosynthetic capacity of the entire PSII. Both Fv/Fo and Fv/Fm demonstrated changes in the efficiency of light energy conversion during photosynthesis under drought-stress conditions. The alterations in these indicators confirmed that the plants were experiencing a water deficit and were under drought stress by day 12 of the drought treatment. 

Chlorophyll a is a key pigment that is involved in multiple chlorophyll-protein complexes within plants’ photochemical and carbon fixation systems [32]. It is instrumental in capturing light energy during photosynthesis. Conversely, chlorophyll b is primarily used in the creation of light-harvesting chlorophyll-protein complexes within the photochemical system. These complexes assist in absorbing light energy and transferring it to chlorophyll a, thereby facilitating the photosynthetic process. Despite variations based on plant characteristics and conditions, chlorophyll content serves as an effective indicator of desiccation tolerance. A common trait under drought stress conditions is a decrease in leaf chlorophyll content. This reduction in chlorophyll levels is often linked to oxidative stress and chlorophyll damage. The chlorophyll content is closely linked to photosynthetic activity, and alterations in chlorophyll levels can significantly affect a plant’s overall photosynthetic performance. Thus, monitoring chlorophyll content offers valuable insights into a plant’s physiological response to drought stress and its photosynthetic activity. In this study, chlorophyll content did not decrease under drought treatment conditions until day 12, and it increased in the case of the 72-31 clone (Table 1). The chlorophyll content after the drought treatment differed in 72-30, with only the chlorophyll a/b ratio increasing. In contrast, the contents of chlorophyll a, b, total, and carotenoids all increased in 72-31, while the a/b ratio remained unchanged. The increase in the a/b ratio of 72-30 is attributed to a slight increase in a, which is anticipated to enhance the activity of the photochemical and carbon fixation systems. Coupled with the fluorescence response of Fv/Fm, this suggests that the drought stress conditions were not severe enough to significantly reduce photosynthesis. Furthermore, our previous study results corroborated that the *Populus alba × Populus davidiana* hybrid clone exhibited minimal changes in chlorophyll content even under drought stress [32]. This also implies that clone 72-31 possesses superior chlorophyll retention capacity under water-deficit conditions.

Plants increase the concentration of soluble sugars to maintain cell turgor under drought-stress conditions. This improves the ability of the cell to retain and absorb water [33,34]. Soluble sugars play a critical role in maintaining the osmotic balance of plants under drought stress [35,36], and this can be an indicator to identify plants that lack water [37]. Glucose and sucrose serve as substrates for osmolytes and cellular respiration in plants, helping to maintain cell homeostasis [31]. Fructose, in contrast, is not directly involved in osmoprotection but is associated with the synthesis of secondary metabolites [38]. It is generally observed that drought stress leads to an increase in soluble sugars, which aligns with our results (Table 2) [39]. While many plants typically exhibit an increase in sugar content during starch degradation under drought stress [35], the sucrose content remained stable in clone 72-31 and increased in clone 72-30. Both clones showed increased glucose and fructose content at day 12 of drought treatment. However, only the 72-30 clone had a significant increase in sucrose, while the 72-31 clone had a significant decrease in starch but unchanged sucrose content. These results indicate that the 72-30 clone had a more severe intracellular water deficit. It was confirmed that the 72-30 clone reacted more sensitively than the 72-31 clone in terms of the change in soluble sugars under drought stress.

Proline is an amino acid that builds up in response to a variety of environmental factors, including water scarcity, salinity, low temperature, and heavy metal accumulation. Additionally, proline is a crucial variable amino acid in regulating the architectures of proteins and membranes, as well as scavenging reactive oxygen species (ROS) in drought-stressed organisms [40]. Proline content increased in both clone 72-30 and clone 72-31, especially in clone 72-30 (Figure 2h). Despite the increase in proline content in both clones, the pattern of gene expression was different. LBO1 and LOC118040847 (DMR6-LIKE OXYGENASE 2-like) were significantly up-regulated and LOC118060471 (1-aminocyclopropane-1-carboxylate oxidase 5-like) and LOC118034855 (probable 2-oxoglutarate-dependent dioxygenase AOP1) were significantly down-regulated in clone 72-30 (Figure 6). In contrast, in the 72-31 clone, the above genes did not change, whereas the expression levels of LOC118037479 (probable 2-oxoglutarate-dependent dioxygenase) and LOC118049366 (1-aminocyclopropane-1-carboxylate oxidase homolog 1-like) were greatly reduced. ABA plays a pivotal role in responses to abiotic stress, such as drought stress [41,42,43,44]. Abiotic stresses increase ABA concentrations in plants by regulating genes involved in ABA synthesis. ABA is synthesized in several steps from beta-carotene, which itself is involved in regulating the calcium signaling pathway, which in turn regulates ABA synthesis and degradation. Increased ABA levels induce the activity of stress response genes, which regulate the stomatal size of guard cells to prevent dehydration [45]. The genes involved in ABA synthesis (NCED3, NCED6, CYP707A2, CYP707A4, BETA-OHASE 1, D27, CCD7, CCD8, PSY) all belong to the carotenoid synthesis pathway (Figure 8). Carotenoids are also used as precursors for the synthesis of phytohormones such as ABA. Therefore, carotenoids are also heavily involved in and regulate plant growth development and stress response [46]. Clone 72-31 had increased carotenoid content, and all genes except NCED6 and CYP727A2 had significant changes in expression (Table 1) (Figure 8). Carotenoids prevented lipid damage by preventing peroxidative damage, which was associated with lower MDA content (Figure 2b). Carotenoid levels increased significantly in clone 72-31 and were maintained in clone 72-30. Carotenoids have been found to possess antioxidant properties, enabling them to scavenge reactive oxygen species such as singlet oxygen and lipid peroxyradicals. They also inhibit lipid peroxidation and scavenge superoxide radicals, which help protect cellular components and maintain cellular integrity during dehydration [47]. 

Clone 72-30 also had significantly increased soluble protein, MDA, and H_2_O_2_ contents (Figure 2). This indicates that clone 72-30 experienced more severe drought stress. Changes in protein levels in plants have also been identified as an indicator of drought stress [48]. While soluble protein levels generally decrease under drought stress due to oxidative stress, there have been reports of increases in response to rapid drought stress in certain plants [35,49,50]. This result is also likely related to the increased levels of H_2_O_2_, decreased SOD, and decreased POD in the 72-30 clone. Antioxidant enzymes have been reported to alleviate the oxidative stress caused by drought stress [51]. In particular, SOD plays an important role in antioxidant defense and redox regulation [52]. The SOD content decreased only in 72-30. In general, it is known that SOD is responsible for converting more reactive and stronger ROS into less reactive and weaker H_2_O_2_, and in many plants, SOD activity increases under drought stress. However, in our results, it was lower in clone 72-30. It has been reported that Fe-SOD is sensitive to the H_2_O_2_ concentration [50,53]. A related gene, FSD2, showed increased expression in both 72-30 and 72-31 clones in our transcriptomic analysis. The H_2_O_2_ content was measured to assess the plant’s ability to scavenge free radicals under oxidative stress [54]. In particular, the increase was greater in clone 72-30, which correlated with a significant increase in the amount of H_2_O_2_. These results are consistent with our previous work on the drought response in other poplar species, which also demonstrated significant reductions. This suggests that antioxidant enzymes, particularly SOD, may not be effectively scavenging ROS in the initial response to drought stress in poplar seedlings. Therefore, future research should explore enhancing SOD expression for the development of drought-tolerant or abiotic stress-resistant poplar cultivars. The increase in H_2_O_2_ content is likely due to increased photosynthesis or photorespiration rather than a result of SOD activity [55]. It is well known that the byproducts of photosynthesis and photorespiration account for a very large proportion of H_2_O_2_ production. 

POD levels decreased in both clones 72-30 and 72-31 (Figure 2). Plant peroxidases, which exist in several gene families and take many forms, have been linked to various functions in plants, including the degradation of H_2_O_2_ and many stress-related responses [56]. However, in this study, despite an increase in H_2_O_2_ content, the drought stress response of poplar’s POD activity decreased in clone 72-30. Transcriptome analysis revealed a slight increase in the expression of PRX52, a peroxidase-related gene, in clone 72-30, while PER4 significantly decreased in both clones. Regarding CAT, both clones 72-30 and 72-31 exhibited an increase compared to the control, with a more pronounced increase in 72-31. Conversely, APX expression significantly increased only in clone 72-31, more than doubling. These results also corresponded to the increased H_2_O_2_ in clone 72-30. APX and CAT are crucial in the final conversion of H_2_O_2_ into water and hydrogen. APX, which is typically more active and important, is found in the chloroplast, mitochondria, and cytosol, where it regulates steady-state H_2_O_2_ concentrations. CAT, in contrast, is located in the peroxisome and aids in the removal of H_2_O_2_ produced by excessive stress. Therefore, based on the findings of this experiment, it is plausible that clone 72-31 has higher APX activity, particularly under drought stress conditions. This could be one of the reasons why it exhibits greater resistance to drought stress.

Drought stress can lead to oxidative damage in plants due to the accumulation of ROS, such as H_2_O_2_, superoxide, hydroxyl radical, and singlet oxygen. These ROS can cause the peroxidation of unsaturated fatty acids in phospholipids, resulting in damage to the cell membrane and macromolecules under both abiotic and biotic stress conditions [57]. The drought-treated clone 72-30 showed a nearly threefold increase in MDA content, which was associated with an increase in H_2_O_2_ content and a decrease in SOD and POD activity (Figure 2). The ROS scavenging function of antioxidant enzymes was weakened after drought treatment, but no decrease in the content of chlorophylls was observed, suggesting that ROS accumulation was accelerated and induced cell damage.

The antioxidant response of poplar to drought stress was not characterized by increased activity of antioxidant enzymes, as in many plants. Taken together with the results of previous studies [58], it can be inferred that poplar has weak antioxidant activity at the onset of drought stress. Further studies should look at changes in antioxidant enzymes after prolonged or intense drought stress.

## 4. Materials and Methods

### 4.1. Plant Materials and Drought Stress Treatment

In this study, *P. alba* × *P. glandulosa*, which are widely distributed and adaptable, were used, and the *P. alba* × *P. glandulosa* hybrid clones 72-30 and 72-31 were made by the National Institute of Forest Science (NIFoS) in Korea (37°15′04″ N, 136°57′59″ E). The *P. alba* × *P. glandulosa* clones 72-30 and 72-31 were selected by NIFoS and have excellent growth characteristics. We conducted this study to observe the tolerance of these two clones to abiotic stress, specifically their response to drought stress. The experiment was carried out over a period of 14 weeks at NIFoS in Suwon under semi-controlled conditions. It took about 14 weeks in total, including the growth period after cuttings before the experiment, the greenhouse adaptation period, and the drought stress treatment period. Plants were used for cuttings of the same clones, and three replicates were performed using 9 plants per replicate (control: 3 replicates × 9 plants; drought 3 replicates × 9 plants). The control group was given sufficient water every two days, and the drought treatment group was provided with good drainage and was not supplied with water for 12 days. Poplar seedlings were grown in topsoil in pots (perlite and vermiculite (2:1:1), 23 cm × 23 cm × 23 cm) containing adequate soil moisture. Prior to the drought treatment, we watered the plants until the soil moisture level reached 40%. We then left the plants unwatered for 12 days. Control plants were kept under identical conditions, with the exception that the soil’s relative water content (RWC) was consistently maintained at 40%. We measured soil moisture every other day using a moisture probe (ICT International Pty. Ltd., Armidale, NSW, Australia). Leaf samples were collected on the first day of the experiment (72-30_d1, 72-31_d1), and leaf samples from the drought stress treatment group were collected on the 12th day when the plants were sufficiently stressed under water shortage conditions (72-30_d12, 72-31_d12). Leaves were harvested at the same time in the morning. The upper and lower leaves were harvested and mixed at the same stage, and 9 samples per replicate were pooled and used in the experiment. After 1 and 12 days of treatment, we collected leaf samples, which were immediately frozen in liquid nitrogen and stored at −80 °C until needed. Each treatment included three biological replicates. We divided eighteen pots into control and drought-treatment groups, with nine pots in each group. This resulted in a total of three replicates, each consisting of three pots.

### 4.2. Measurement of Chlorophyll, Soluble Sugars, Starch, Proline, MDA, H_2_O_2_, Soluble Proteins, ABA and Antioxidant Enzymes

In the physiological parameter analysis, leaf samples from nine seedlings per treatment were finely ground under liquid nitrogen. The chlorophyll and carotenoid levels in the leaves were determined using the methods outlined in previous work [59,60]. Soluble sugar and starch were analyzed as previously described in Walters’ method [59,61]. Proline, MDA, and H_2_O_2_ were measured using the methods described in earlier studies [59,62,63,64]. Fresh leaf samples, each containing 0.1 g of plant tissue, were collected in triplicate. 

We used the Bradford assay method and bovine serum albumin as a standard to estimate the concentration of soluble proteins [65]. The absorbance at 595 nm was measured using a Biospectrometer (Eppendorf, Hamburg, Germany). To measure antioxidant enzyme activity, a superoxide dismutase (SOD) assay kit (DG-SOD400, Dogen, Seoul, Republic of Korea) was used, and catalase (CAT) activity was analyzed using a catalase (CAT) assay kit (DG-CAT400, Dogen, Seoul, Republic of Korea). A plant ascorbate peroxidase (APX) kit (MBS2602897, MyBioSource, San Diego, CA, USA) was used to determine APX activity in homogenized leaf samples. A plant peroxidase (POD) ELISA kit (MBS9313803, MyBioSource, San Diego, CA, USA) and plant hormone abscisic acid (ABA) ELISA kit (MBS703081, MyBioSource, San Diego, CA, USA) were used to measure the content of POD and ABA in leaves, respectively. We performed all procedures in accordance with the manufacturer’s protocols. After preparation, we immediately measured the optical density at 450 nm, 560 nm, 450 nm, 450 nm, and 450 nm, respectively, using an automated plate reader (SpectraMax M2, Molecular Devices, San Jose, CA, USA).

### 4.3. RNA Extraction, cDNA Library Construction, Sequencing

Total RNA was extracted from the leaves of three biological replicates of poplar using a Beniprep^®^ Super Plant RNA extraction kit (InVirusTech Co., Gwangju, Republic of Korea). Around 2 g of RNA from each sample was utilized to construct cDNA libraries. The integrity of the isolated RNAs was assessed using the Bioanalyzer 2100 system (Agilent Technologies, Inc., Santa Clara, CA, USA). The Truseq Stranded mRNA Prep Kit (Illumina Technologies, San Diego, CA, USA) was employed for the subsequent step of cDNA library creation, provided the RNA integrity number (RIN) was greater than 7. Following this, the constructed cDNA libraries were sequenced on an Illumina NovaSeq 6000 platform to generate 101 bp paired-end reads. The submission IDs for the raw data sent to the NCBI SRA can be found in Supplementary Table S1.

### 4.4. Assembly Normalization and Quality Assessment

Prior to assembly, poor-quality reads and reads containing adaptor sequences were removed from the raw data of the 12 samples using Trimmomatic software (version 0.39) under default settings [66]. The sequence quality s per base was checked using FastQC (version 0.11.2). Filtering of the Fastq files was then performed. The trimmed reads were then aligned to a reference genome (*P. alba*) using HISAT software (version 2-2.1.0) [67]. To compare reads that had strand information to those that did not, reads were also aligned without the ‘--RNA-strandness RF’ option. The mapped reads were then quantified using FeatureCounts with annotation files (.GFF3) for protein-coding genes [68].

### 4.5. Differentially Expressed Genes (DEGs) Analysis

Transcriptomes of *P. alba* × *P. glandulosa* tissues were examined in pairs: 72-30_d1 (numbers 1, 2, and 3), 72-30_d12 (numbers 4, 5, and 6), and 72-31_d1 (numbers 7, 8, and 9), 72-31_d12 (numbers 10, 11, and 12). Up- and down-regulated DEGs were identified based on a log_2_FC ≥2 or ≤−2, with a *p*-value of 0.05, respectively. A Venn diagram generated in Venny v2.1.0 (https://bioinfogp.cnb.csic.es/tools/venny/; accessed on 3 March 2023) was used to examine DEGs that were universally and uniquely regulated in all tissues. 

### 4.6. Gene Ontology (GO) and Kyoto Encyclopedia of Genes and Genomes (KEGG) Pathway Analysis

The gene symbols for all the transcripts were assigned by performing BLASTX (evalue = 1 × 10^−5^) against the *A. thaliana* reference protein dataset. The best BLASTX matches were assigned to GO classes. GO functional classification was performed using WEGO analysis [69]. Using the gene information of the *P. alba* dataset in the KEGG database, manually determined DEGs involved in proline, abscisic acid, and antioxidant metabolism were identified.

### 4.7. Quantitative PCR (qPCR) Validation of RNA-Seq Data

RNA samples were converted into single-stranded cDNA using the cDNA EcoDry^TM^ Premix (TaKaRa, Kuastsu, Shiga, Japan). Real-time qPCR was performed using a CFX96 Touch Real-Time PCR Detection System (BIO-RAD, Hercules, CA, USA) with IQ^tm^ SYBR Green Supermix (BIO-RAD, Hercules, CA, USA). The following conditions were used for the reaction: 95 °C for 30 s, 38 cycles of 95 °C for 5 s, and 60 °C for 34 s. We ran three independent biological replicates and three technical replicates for each biological replicate. The 2^−ΔΔCt^ method was used to analyze the relative abundance of transcripts [70]. The internal controls were *Actin* and *UBQ7* in *Populus* [71]. The primers for each gene are listed in Supplementary Table S2.

### 4.8. Statistical Analysis

Differences were analyzed through one-way ANOVA with multiple comparisons using Tukey’s HSD. *p* values < 0.05 were considered significant. Values are presented as the mean with standard deviation (SD).

## 5. Conclusions

Several indicators of drought stress, including elevated MDA, H_2_O_2_, proline, and ABA, along with elevated soluble sugars and protein, indicated that clone 72-30 was more stressed than clone 72-31. Water-soluble sugars and proline contents, which contribute to osmotic regulation important for drought stress tolerance, showed similar increases in both clones. However, regarding antioxidant activity, only clone 72-31 showed a significant increase in the activities of APX and carotenoids under drought stress, which may have led to the lower levels of H_2_O_2_ and MDA in this clone. Therefore, it seems that differences in antioxidant enzyme activity are more accountable for the variations in drought stress response between these two hybrid poplars than their osmotic regulation capabilities. The superior drought tolerance indicators of clone 72-31 may be linked to its enhanced activity of APX and carotenoids, both enzymatic and non-enzymatic antioxidants. Further research is required to explore ways to boost antioxidant activity for the selection and enhancement of poplar seedlings with exceptional tolerance. Additionally, through transcriptome analysis, we verified that the two poplar clones exhibited changes in their genetic response to drought stress. Specifically, we observed alterations in genes related to the synthesis of proline, ABA, carotenoids, and both enzymatic and non-enzymatic antioxidants. As the two clones responded differently to drought treatment, variations in gene expression were also noted. Therefore, further research on these genes will aid in understanding the mechanism of drought stress in poplar. The findings of this study are anticipated to assist in the selection of more drought-tolerant *Populus* clones and in studies of seedling drought tolerance.

## Figures and Tables

**Table 1:** Effect of drought stress treatment on the photosynthetic pigments in *P. alba* × *P. glandulosa*.

Clone	Treatment	mg/g FW				Chl a/b	Chl/Car
		Chl a	Chl b	Total Chl	Carotenoids		
72-30	Control	1.76 ± 0.09 ^ab^	0.73 ± 0.04 ^a^	2.49 ± 0.31 ^a^	0.53 ± 0.02 ^a^	2.42 ± 0.07 ^b^	4.70 ± 0.11 ^n.s^
	Drought	1.92 ± 0.07 ^a^	0.72 ± 0.04 ^ab^	2.68 ± 0.20 ^a^	0.55 ± 0.02 ^a^	2.68 ± 0.16 ^a^	4.84 ± 0.01 ^n.s^
72-31	Control	1.51 ± 0.10 ^b^	0.64 ± 0.05 ^b^	2.14 ± 0.15 ^b^	0.45 ± 0.02 ^b^	2.38 ± 0.06 ^b^	4.81 ± 0.11 ^n.s^
	Drought	1.91 ± 0.15 ^a^	0.76 ± 0.05 ^a^	2.68 ± 0.20 ^a^	0.57 ± 0.05 ^a^	2.51 ± 0.07 ^ab^	4.68 ± 0.13 ^n.s^

The values are the means ± SD (*n* = 3). Different lowercase letters indicate significant differences, and “n.s” indicates not significant (ANOVA with Tukey’s HSD, *p* < 0.05).

**Table 2:** Effect of drought stress treatment on carbohydrate content in *P. alba* × *P. glandulosa*.

Clone	Treatment	mg/g FW			
		Glucose	Fructose	Sucrose	Starch
72-30	Control	1.18 ± 0.10 ^d^	1.00 ± 0.11 ^c^	54.70 ± 1.20 ^b^	0.28 ± 0.11 ^ab^
	Drought	2.46 ± 0.25 ^b^	2.69 ± 0.04 ^a^	82.38 ± 2.45 ^a^	0.28 ± 0.08 ^ab^
72-31	Control	2.10 ± 0.13 ^c^	2.03 ± 0.25 ^b^	51.51 ± 3.97 ^b^	0.37 ± 0.12 ^a^
	Drought	2.92 ± 0.22 ^a^	3.01 ± 0.42 ^a^	49.83 ± 5.30 ^b^	0.19 ± 0.05 ^b^

The values are the means ± SD (*n* = 3). Different lowercase letters indicate significant differences (ANOVA with Tukey’s HSD, *p* < 0.05).

**Table 3:** Summary of RNA sequencing data.

Sample	Raw Reads	Clean Reads	Mapped (%)	Q30 (%)	GC (%)
72-30_d1_1	21,322,525	19,423,209	91.94%	95	47
72-30_d1_2	25,534,053	23,613,885	92.22%	95	48
72-30_d1_3	27,750,144	24,773,305	84.14%	95	49
72-30_d12_1	29,270,661	26,337,948	90.26%	95	47
72-30_d12_2	29,362,659	26,669,548	89.28%	95	48
72-30_d12_3	32,321,191	29,446,757	88.63%	95	46
72-31_d1_1	28,614,343	26,106,663	86.77%	95	47
72-31_d1_2	32,949,652	29,442,606	89.40%	95	46
72-31_d1_3	32,479,330	30,465,003	88.08%	95	48
72-31_d12_1	30,944,699	29,702,711	85.40%	95	48
72-31_d12_2	29,104,362	27,656,786	81.95%	95	49
72-31_d12_3	27,334,799	26,081,336	80.33%	95	50
Sum	346,988,418	319,719,757			
Mean	28,915,701.5	26,643,313.08	87.37%	95	47.7

**Table 4:** Log-fold changes of top 10 down-regulated DEGs and top 10 up-regulated DEGs in 72-30 d1 versus 72-30 d12.

Gene ID	Gene Description	Log2 FC	*p*-Value	TAIR ID	Gene Symbol	Regulation
LogFC > 0						
LOC118043229	galactinol synthase 2-like	12.27314	1.13 × 10^−13^	AT2G47180	*GolS1*	Up
LOC118043085	bidirectional sugar transporter SWEET15-like	11.40774	2.21 × 10^−21^	AT5G13170	*SAG29*	Up
LOC118053155	zeatin O-xylosyltransferase-like	10.90792	2.54 × 10^−18^	AT2G15490	*UGT73B4*	Up
LOC118057602	uncharacterized protein	10.08275	5.60 × 10^−11^	AT5G50360		Up
LOC118033421	embryonic protein DC-8	9.94831	1.14 × 10^−14^	AT2G36640	*ECP63*	Up
LOC118062093	dehydration-responsive element-binding protein 2D-like	9.40484	5.60 × 10^−13^	AT1G75490		Up
LOC118052105	umecyanin-like	9.22683	2.11 × 10^−11^	AT2G25060	*ENODL14*	Up
LOC118061594	non-specific lipid-transfer protein-like protein	9.08816	6.39 × 10^−6^	AT3G22600		Up
LOC118034863	late embryogenesis abundant protein D-7	9.00364	2.88 × 10^−13^	AT3G15670		Up
LOC118055598	glucose and ribitol dehydrogenase-like	8.91445	9.97 × 10^−28^	AT1G54870		Up
LogFC < 0						
LOC118040537	auxin-induced protein 15A-like	−5.69993	0.0013	AT4G34770		Down
LOC118059218	uncharacterized protein	−5.49169	0.0040	AT4G04745		Down
LOC118047630	acetyl-CoA-benzylalcohol acetyltransferase-like	−5.44358	0.0007	AT3G26040		Down
LOC118039754	protein RADIALIS-like 2	−5.42122	0.0039	AT1G75250	*RL6*	Down
LOC118044774	uncharacterized protein	−5.39230	0.0065	AT4G12690		Down
LOC118043148	protein HEADING DATE 3A-like	−5.24861	0.0306	AT1G65480	*FT*	Down
LOC118063338	syntaxin-112-like	−5.18643	0.0395	AT2G18260	*SYP112*	Down
LOC118053660	uncharacterized protein	−5.17855	0.0099	AT3G01410		Down
LOC118042489	cyclin-U4-1-like	−5.16138	0.0385	AT2G44740	*CYCP4;1*	Down
LOC118058270	RNA exonuclease 4-like	−5.13317	8.59 × 10^−5^	AT3G27970		Down

DEG—differentially expressed gene; FC—fold change.

**Table 5:** Log-fold changes of top 10 down-regulated DEGs and top 10 up-regulated DEGs in 72-31 d1 vs. 72-31 d12.

Gene ID	Gene Description	Log 2FC	*p*-Value	TAIR ID	Gene Symbol	Regulation
LogFC > 0						
LOC118057602	uncharacterized protein	8.97992	7.9 × 10^−19^	AT5G50360		Up
LOC118056377	probable protein phosphatase 2C 75 isoform X1	7.09109	4.6 × 10^−6^	AT5G51760	*AHG1*	Up
LOC118028052	WUSCHEL-related homeobox 2-like	6.87533	2.2 × 10^−5^	AT5G59340	*WOX2*	Up
LOC118043229	galactinol synthase 2-like	6.48803	3.5 × 10^−61^	AT2G47180	*GolS1*	Up
LOC118047047	uncharacterized protein	6.37664	4.0 × 10^−9^	AT1G07985		Up
LOC118044732	uncharacterized protein	6.14501	5.2 × 10^−31^	AT5G50360		Up
LOC118032224	uncharacterized protein	6.08772	6.9 × 10^−4^	AT4G33467		Up
LOC118046914	translocator protein homolog	6.07722	6.3 × 10^−6^	AT2G47770	*TSPO*	Up
LOC118053986	putative disease resistance RPP13-like protein 1	5.97234	9.5 × 10^−4^	AT3G14470		Up
LOC118029876	heavy metal-associated isoprenylated plant protein 27	5.91057	4.6 × 10^−3^	AT5G66110	*HIPP27*	Up
LogFC < 0						
LOC118058995	auxin-responsive protein SAUR24-like	−6.84085	2.3 × 10^−6^	AT5G18020	*SAUR20*	Down
LOC118061976	ornithine decarboxylase-like	−6.12173	1.2 × 10^−4^	AT5G11880		Down
LOC118056925	uncharacterized protein	−5.77476	8.6 × 10^−4^	AT3G15115		Down
LOC118045856	major allergen Pru ar 1-like	−5.69965	1.3 × 10^−3^	AT1G24020	*MLP423*	Down
LOC118039754	protein RADIALIS-like 2	−5.61201	1.1 × 10^−3^	AT1G75250	*RL6*	Down
LOC118044774	uncharacterized protein	−5.61180	1.1 × 10^−4^	AT4G12690		Down
LOC118035984	axial regulator YABBY 1-like	−5.56260	1.4 × 10^−3^	AT2G45190	*AFO*	Down
LOC118051349	probable serine/threonine-protein kinase PBL7	−5.48302	4.1 × 10^−4^	AT1G54820		Down
LOC118028545	leucoanthocyanidin dioxygenase-like	−5.46618	2.9 × 10^−5^	AT4G22880	*LDOX*	Down
LOC118055368	terpene synthase 10-like	−5.45600	3.3 × 10^−2^	AT3G25830	*TPS-CIN*	Down

DEG—differentially expressed gene; FC—fold change.

## Data Availability

The data presented in this study are available on the NCBI SRA database (PRJNA989398, accessed on 30 June 2023).

## Supplementary Materials

The following supporting information can be downloaded at https://www.mdpi.com/article/10.3390/plants12183238/s1, Table S1: Summary of Sequencing information and mapping quality information; Table S2: Sequence of primers used for qPCR in *P. alba × P. glandulosa*.
