# Supplementary material for: Transcriptomic and Physiological Analysis Reveals Genes Associated with Drought Stress Responses in Populus alba × Populus glandulosa
Source: Plants (Basel). 2023 Sep 12;12(18):3238. doi: 10.3390/plants12183238 (PMC10535988; doi:10.3390/plants12183238)
Supplement: Supplementary file 1 [file plants-12-03238-s001.zip › Table S2.pdf]

Supplementary Table 2. Sequence of primers used for qPCR in *P. alba* × *P. glandulosa*

Gene ID	Gene Symbol	Forward primer (5’→3’)	Reverse primer (5’→3’)
LOC118062568	*SAG12*	TCTGTTGCTGTTGACGGTGG	TTTCACCCCAACTGGTTCCC
LOC118032010	*SLAH1*	TGCGAGAAATTCGGAACCCG	CCGGCATGTAACCTAGCCAA
LOC118052105	*ENODL14*	CCTGCCAGTCCTGTCACATT	GCCCTTACAAGCCTACCTGG
LOC118053155	*UGT73B4*	GGCCACCGGCTTTCTCATTA	GTGTCGGGGGTGGGTTTTAT
LOC118043148	*FT*	GACGGAGGACAAACCCATCT	GTAGCGATCTCGATACCTACAAT
LOC118042489	*CYCP4.1*	CCTCCAGTCTCCTCTCTCGT	GAACCGATCGAGTCAACCCA
LOC118029876	*HIPP27*	GGTACGTCCGAAATCCGCTT	CACAACTCATCAATCACAGGCG
LOC118055294	*ROXY2*	TGCTGAGTCCTTACACACAC	GCTGCTGCTTTGGAGTATTGG
LOC118035984	*AFO*	GAATAACGTGCGCCAACAGG	CTTGACGGACGTCTGAACCT
LOC118038286	*NCED3*	CTCAAATCTCACACTGGGACT	GAGGCTACCCTCTCCTTTTCC
LOC118035767	*CYP707A4*	TCATAAGCTGCTGAGATGCCA	ATGGGCCTTGGGAGAGGATA
LOC118030260	*β-OHASE 1*	ATGGAGTCAGGAATCACCGC	GGAAGGGACAGTGAGGTTGT
LOC118035629	*PRX52*	GTTCCTTGTGTTGGCTGTTCC	CGCATCACAACCATGGACAAA
LOC118060062		GGATTTGCAGTGCCGTCAAC	GCACACTTTATTAAGCGAATGCAC
LOC118054546	*TT4*	GTCGAGTGCATGCGTGTTGT	GCAACGGACGCTGCTTCT
LOC118058446		AAAACGCGTCAAGAGTTCGC	GGGGCCAGAGAAATTACCACT
LOC118040847		GATGCTGTAATCGGGCCTCC	TGCAACTGAAATCTTGCCCC
LOC118034519	*LBO1*	CCACTGGGCATTAACAATGCAA	GAAACGGGAACAGGAGCCAT
	UBQ7	GGAACGGGTTGAGGAGAAAGAAG	GCAAGAACAAGATGAAGCACAGAGC
	ACTIN	TTCTACAAGTGCTTTGATGGTGAGTTC	CTATTCGATACATAGAAGATCAGAATGTTC
